# Menstruation among autistic adults: An occupational perspective

**DOI:** 10.1177/03080226251341730

**Published:** 2025-05-26

**Authors:** Samantha LJ Bowden, Paul K Miller

**Affiliations:** University of Cumbria, Lancaster, UK

**Keywords:** Autism, menstruation, qualitative, adult, occupation

## Abstract

**Introduction:**

Menstruation is known to have potentially adverse impacts at multiple levels of occupational performance. However, little research has directly investigated the everyday menstruation experiences of autistic individuals, for whom menses and menarche are widely thought to be particularly occupationally disruptive.

**Method:**

A qualitative research design was employed to address the lived experiences of menstruation among *N* = 6 autistic adults living in the United Kingdom. With institutional ethical approval, in-depth online interviews were conducted, yielding 34,734 words of transcript. Thematic analysis of these revealed interconnected global themes.

**Findings:**

The global themes identified were: (1) ‘Sense of self’, addressing participants’ sensory overload and experiences of anxiety, ‘brain fog’ and concern with cleanliness. (2) ‘Attributional work’, addressing events and contexts which were taken to trigger and/or exacerbate key problems (such as the need to use public restrooms). (3) ‘Reclaiming orderliness’, addressing participants’ pragmatic strategies for overcoming increased unpredictability in their lives during periods.

**Conclusion:**

Menstruation poses specific, significant challenges for autistic individuals that require autism-specific solutions. These challenges impact individuals’ ability to perform occupations of self-care, productivity and leisure. As such, Occupational Therapists have a key role in the provision of support to address the impact of menstruation on occupational engagement and participation.

## Introduction

Recent academic discourse and research have largely focused on biomedical outcomes of the interaction between menstruation and autism. Contemporary studies in this domain converge on an understanding that menstruation presents autistic individuals with a greater prevalence of dysmenorrhoea and/or menorrhagia, plus mood, behaviour and/or hygiene issues than is generally seen in neurotypical populations (Jeffery et al., 2013; Steward et al., 2018). In addition, the increased prevalence of severe premenstrual syndrome in autistic individuals has been associated with a deviation in hormonal fluctuations from those seen outside of the autistic community (American Psychiatric Association, 2013; Obaydi and Puri, 2008). Research has, to date, also determined that the experience of menstruation often negatively impacts individuals in three key features of occupational performance across a range of populations; self-care, productivity and leisure (Ballan and Freyer, 2017; Corscadden and Cassely, 2021). At the time of writing, however, there exists a limited body of research directly addressing menstruation and autism from an occupational perspective, and a smaller literature still proceeds from the perspectives of autistic individuals themselves (Steward et al., 2018).

This paper reports a qualitative interview-based study of the menstruation experiences of autistic adults in the United Kingdom (UK), aiming to specifically elucidate how menstruation might cause highly significant – but nevertheless routine – challenges for occupational performance among autistic individuals. It is contended that the findings, although emergent of a relatively small and localised sample, might nevertheless (a) help accentuate and illuminate a phenomenon that has previously occupied very limited space in pertinent public and academic discourse, (b) lay down some foundations for further research and (c) inform prospective thinking around day-to-day occupational therapy work in a domain where there was not previously a robust evidence base for best practice.

## Literature review

Although the experience of autism varies greatly between individuals in both presentation and impact, all cases are clinically characterised by (a) difficulties with socio-communicative interaction and (b) restrictive and/or repetitive (or otherwise unusual) sensory-motor behaviours (American Psychiatric Association, 2022; World Health Organization, 2019). On a more vernacular level, autism is also commonly associated with emotional regulation challenges and altered sensory sensitivities (George and Stokes, 2018; Hull and Mandy, 2017). Diagnosis is typically based on socio-behavioural observation and developmental history and is often made within the first 2 years of an individual’s life, although many individuals are diagnosed at significantly more advanced ages (Lord et al., 2018).

It should be noted at this point that clinical definitions of autism are natively born of a classically medical or ‘deficit’ model of health and illness, and it is unequivocally the case that autism can be deeply disenabling in a proportion of affected individuals. ‘Disabled’ is demonstrably not, however, how all autistic individuals experience themselves. Indeed, autistic self-identification has been strongly associated with a broader view of autism as a positive identity, and certainly not an illness that requires ‘curing’ (Kapp et al., 2013). This paper, therefore, proceeds from an understanding of autism as a way of being, not as an inherent disability, unless otherwise determined in that sense by an involved autistic individual.

### Autism and menstruation: Issues of prevalence

The worldwide prevalence of autism remains unclear, largely due to high variability within and across socio-demographic groups, plus strong inequalities between healthcare systems’ capacities for delivering a diagnosis and aggregating diagnostic information (World Health Organization, 2013). The most robust contemporary data, however, indicate that around 1% of children are now diagnosed with autism globally, at an approximate 4:1 male-to-female ratio of incidence (Zeidan et al., 2022). In the UK, the site of the current study, the current rate of diagnosis among children is thought to be higher, at 1.76% (Roman-Urrestarazu et al., 2021), while around 1.1% of the adult population is estimated to be autistic, with around three to four times as many males diagnosed as females (Buckley, 2017).

Any reported gap between rates of diagnosed autism in children and adults in healthcare systems such as those in the UK is, today, seldom thought to speak to any longitudinally rising incidence of autism itself. Rather, it is generally accepted to indicate a historical pattern of general under-diagnosis (and later-in-life diagnosis), attempts at redressing which are now being made through improved understanding and early recognition (Stagg and Belcher, 2019). Moreover, it has been widely demonstrated that many autistic adults, particularly autistic women, progressively become experts in ‘masking’ (or ‘camouflaging’) their autistic behavioural traits. Masking amounts to a battery of interpersonal stigma-management practices in which learned/copied behaviours are utilised to conceal publicly inferable ‘differences’ (Cooper et al., 2022; Hull et al., 2020; Miller et al., 2021). While this can delay or prevent recognition/diagnosis, it has also been noted to functionally become an occupation in itself (Cage and Troxell-Whitman, 2019; Franklin et al., 2024), somewhat inevitably at the cost of performance in others, and with routine detriments to mental health (Evans et al., 2024).

Given the above, it is reasonable to surmise that among diagnosed autistic adults, the core population of interest in this study, it is not only likely that menstruation itself will present (and will have presented) a particular challenge to occupational performance for impacted individuals through a variety of mechanisms (Armour et al., 2019; Jeffery et al., 2013; Obaydi and Puri, 2008; Steward et al., 2018), but the journey to an autism diagnosis and proceeding support may also have had significant occupational consequences. Furthermore, there is likely to remain a sizeable population of pertinent individuals who remain undiagnosed, and thereby bereft of any targeted help. If Occupational Therapists (henceforth OTs) are to be suitably equipped to work effectively with all such individuals, therefore, research specifically highlighting their practical experiences must be an essential precursor.

### Autism and menstruation: Issues of experience

Many of the known menstruation-related biomedical impacts on autistic individuals have been forestated (American Psychiatric Association, 2013; Jeffery et al., 2013; Obaydi and Puri, 2008). While some recent research around autism has addressed specific impacts of menstruation on lived experience of dysmenorrhoea (Gray and Durand, 2023), and management of neurodiversity and menstrual health at work (Sang et al., 2024), there remains limited direct investigation of how menstruation may affect autistic adults in their routine occupational activities. While a small number of recent primary studies have tangentially engaged the latter while investigating other phenomena related to autism or menstruation (e.g. Cooper et al., 2022; Jeffery et al., 2013), research manifestly addressing it remains limited. At the time of writing, the located literature of this order amounted to a single paper (Steward et al., 2018), which the authors describe as a (largely qualitative) ‘brief online survey’ of 237 post-menarcheal autistic and non-autistic respondents (p. 4278). In said research, it should be noted that many of the pertinent experiences of autistic respondents were not found to deviate greatly from those of their more neurotypical counterparts. Both groups emphasised that they had, at least initially, needed more carefully delivered education/information (mostly from family and schools, though some from other sources) around menarche and the subsequent experience of menstruation. More specifically highlighted was the need to be told about the ‘normal and natural’ character of periods, the variety of specific ways they could affect the body and mind of an individual, and the importance of individuals coming to understand what is and is not normal *for them*. Respondents widely viewed that practical and unvarnished guidance about ‘what to expect’ is critical in helping reduce the general stigma, personal confusion and the incidence of embarrassing events that many of them had experienced themselves.

In terms of the specific experiences of autistic respondents, Steward et al. (2018) highlight key routine challenges, most notably in the domains of sensory hypersensitivity and emotional/behavioural regulation, as often reported to be intensified before, during and after menses. This intensification could, in turn, result in heightened social anxiety, increased incidence and severity of meltdown and, at worst, interpersonal ‘shutdown’. All such outcomes were typically reported to end in partial or total withdrawal from productive engagement on many occupational fronts; professional, social and personal.

It would be uncontroversial to propose that the method employed by Steward et al. (2018) places inherent limits on any claim to describe nuanced and/or well-contextualised participant experience. Although illuminating several important aspects of autistic experiences of menstruation, and like most survey-based approaches that yield some degree of textual evidence, it negates a researcher’s opportunity to coordinatively explore and develop upon a point with a participant (Hopkins and Miller, 2023). Such limitations are, however, well-recognised by the authors themselves, and their robust-yet-modest approach to their data nevertheless lays important substantive foundations for further work in the domain.

## Method

To facilitate a nuanced understanding of the menstruation experiences of autistic individuals, semi-structured interviews were employed to maintain an appropriate focus on the core research topic while also allowing sufficient flexibility for substantive novelties to arise (Waring et al., 2018). With respect to emergent data, an investigative model in line with the reflexive thematic analysis (henceforth RTA) process outlined by Braun and Clarke (2006, 2019) was adopted, given its practicality and established utility within domain-relevant research. Full ethical approval for this study was granted by the pertinent panel at the lead author’s academic institution (reference: 2511/SRPM/2021), and all stages of its conduct were managed in strict accordance with the conditions stipulated.

### Participants, recruitment and ethics

Inclusion criteria for participation were set as follows: participants must have (a) a formal autism diagnosis, (b) personal experience of menstruation, and (c) be 18 years or over at the point of interview. These criteria were published in an invitation on the research webpage of the Autism and Asperger’s Network, and links to this page were shared via a variety of social media. This amounted to a purposive convenience sampling model. Of those who responded to the original call, *N* = 14 requested further information and *N* = 6 completed and returned a signed consent form within the available timescale. All who consented were then interviewed online. Conditions of participation mandated that no participant would need to disclose any demographic variables before their interview, and that any such aspects of identity would only be raised in the reportage of the findings if made actively relevant by participants themselves.

### Procedure

An interview schedule encouraging an open narrative on participants’ experiences of menstruation, with prompts derived largely from the findings of Steward et al. (2018), was prepared by both authors and checked for participant sensitivity by an autism advocate. This schedule was then administered online by the first author, using iterative interviewing techniques where pertinent. Transcribed data were redacted of key identity-sensitive information (names, places, and exact dates) prior to analysis. The resultant corpus of data amounted to 34,734 words. Pseudonyms, chosen by the participants, are used throughout to protect identities.

### Data analysis and rigour

The six stages of Braun and Clarke’s (2006) thematic analysis model were employed to facilitate a systematic investigation of the collected data. In terms of procedural rigour, Table 1 demonstrates in granular terms how triangular consensus validation (Patton, 1990) on the final analysis was managed by the authors, SB (a practicing female occupational therapist and early-career researcher) and PM (a veteran male research psychologist). Also incorporated are pertinent aspects of Yardley’s (2000) conditions of rigour, where relevant.

**Table 1.** Thematic analysis steps.

Phase	Steps taken
(1) Familiarisation with data	Each interview was transcribed ‘from scratch’ by SB, with initial notes taken to guide the coding process.
(2) Generation of codes	All relevant data were systematically coded with both descriptive and interpretive codes by SB, with feedback from PM. Codes were then manually labelled using a colour key and linguistic labels in Microsoft Word.
(3) Search for themes	Once an initial list of codes had been collated by SB and checked for consistency by PM, provisional sub-themes and aggregating global themes were generated by both authors.
(4) Reviewing themes	All themes were reviewed by both authors and on two levels. Firstly, all data within each theme were assessed to determine if they fit coherently within that theme. If not, analysis was reshaped until this was the case. Second, each theme was considered in relation to the whole dataset, with thematic maps utilised to assess whether the relationship between each theme reflected the meaning of the dataset. This was a recursive process, conducted by both authors until both steps were satisfied.
(5) Defining and naming themes	An overall narrative of the data was considered in the naming of themes, with each named in such a way that it describes what aspect of the data the theme represents. Each theme was linked to any sub-themes and listed alongside quotes to establish their integrity.
(6) Report writing	The final report was written, and summaries of the research findings provided via email to participants who had opted in.

*Source*: Adapted from Braun and Clarke (2006).

To address Yardley’s (2000) key precepts regarding impact and importance, meanwhile, the provisional findings were presented at an international OT conference; all feedback has been incorporated into the discussion below.

## Findings

Thematic analysis identified three global themes. These were:

1. Sense of self
2. Attributional work
3. Reclaiming orderliness

**Figure 1.**
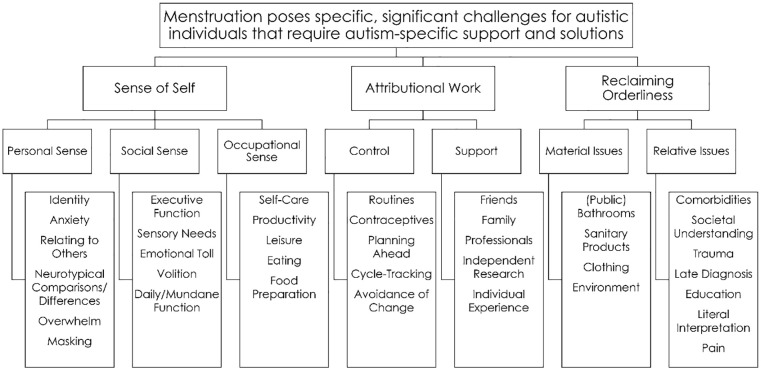
Themes and sub-themes.

The relationship between these and the sub-themes from which they emerged is schematised in Figure 1.

Each theme is described below with reference to direct data. In all accounts given, it should be noted that participants routinely positioned their experiences as entirely consistent with those of other ‘autistics’ or ‘autistic people’ and often counter to that of a nominal ‘neurotypical’ other, with only rare reference to variabilities that might occur within either population. For example:

Elle: Obviously not every single neurotypical, but as a general rule, they haven’t got a clue. They do not understand how much more intense and how much more of an impact [menstruation] has, more on autistics because they don’t live it, they don’t get it at all. We are often just dismissed as exaggerating . . . attention seeking and pity-seeking, but no, this is actually this bad.

Oppositional categories such as ‘neurotypical’ and ‘autistic’ were not actively encouraged in the interview schedule, but frequently emerged in the participants’ narratives nevertheless. This order of accounting does, however, bring into sharp relief the manner in which the participants made sense of their own experience with primary reference to observations of others around them.

### Theme 1: Sense of self

Universally, participants described menstruation as having a negative impact on thoughts and feelings, with issues about potential leakage and (particularly) the capacity to access bathrooms causing complex and enduring anxiety. This anxiety was compounded, in most accounts, by a sense that those around them were not being challenged in the same way.

Becky: Hormones at that time of the month make the average . . . woman with typical PMS more sensitive. When you’re already extremely sensitive anyway regardless of anything to do with periods or hormones, just simply through personality, how you’re made and wired because you’re autistic, then I think it’s definitely a whole new emotional level.

Broadly, this circumstance was compounded by the emotional toll of masking their own experience to fit in with neurotypical friends who faced fewer challenges. This was echoed in other responses, with all feeling their experience was different to, and misunderstood by, the ‘neurotypical’ menstruater. Some, meanwhile, explained how additional cognitive and emotional necessities became overwhelming, contributing to burn-out and meltdowns, while another explained how usual emotional sensitivity would become exacerbated.

Anna: I will just shut down. I will . . . just my brain blocks all the emotions because like it’s too overwhelming. I can’t handle this. It’s like self-preservation for self-protection.

Sensory needs were considered by all participants as significantly challenging to manage when experiencing periods. This ranged from being the most difficult aspect to deal with to a moderate challenge. The use of sanitary products, finding appropriate clothing and the physical sensation of blood loss were identified as aversive sensory experiences. While some participants managed this through pre-planning outfits and having emergency supplies available for regular changing, it was also described how it would often be easier to cancel plans than to adapt to different clothing and routines and further explained that the ‘best’ products for her still result in anxiety and sensory overload.

Fran: Well, when I have sort of sensory overload, I get quite anxious. So then I start breathing really fast. My heart goes fast, my hands feel all sweaty and clammy. Yeah, so it sounds like quite a sort of severe reaction to like having to change a pad but for me that’s just the reality.

Sensory needs were often associated with executive function, contributing to a level of overwhelm resulting in tasks that would usually be manageable becoming much more challenging. A key difficulty related to initiating tasks, and a brain fog which made completing activities much harder.

Claire: The executive dysfunction kicks in a lot sooner when things are at a much lower level, so when it’s much simpler stuff. For example, like cooking a simple meal is a lot harder to do when I’m on my period because the executive dysfunction is exacerbated.

For some participants, completing tasks with long sequences becomes overwhelming, resulting in a lack of motivation to carry out daily tasks. Despite differences in the presentation of the impact on executive function, an impact was shared by most participants.

Becky: I find information processing tough at the best of times . . . This becomes so much harder at that time of the month. Just total brain fog and cloudiness with a looming period makes trying to concentrate and focus on anything and process information difficult.

All described consequent changes to participation in their occupations, each having cancelled leisure activities while menstruating, and associated decreases in productivity, were mentioned by most. Self-care activities were universally impacted, with two identifying food preparation and eating as problematic. A key attribution was that the steps required to prepare food become overwhelming, leading to reliance on pre-prepared meals. Furthermore, while some find showering and bathing alleviates discomfort, others find maintaining hygiene increasingly difficult, owing to sensory experience, reduced volition and the challenge to accommodate further demands on executive function. While one participant described feelings of uncleanliness and utilised self-care to cope, another acknowledged the need to maintain hygiene; however, finds this more taxing when experiencing sensory aversions to blood.

Fran: You never quite know if somethings gonna come out and you’re gonna see blood when you weren’t expecting it. . . I just don’t like the feeling and I don’t like the way it looks. So, I’ll do it (shower) because I have to, but not because I want to.

Dani: I always felt really, really unclean. I would take extra baths, I would take extra showers.

### Theme 2: Attributional work

Unpredictability was identified by all participants as a significant stressor, with routines being considered important for executive functioning and self-regulation. Most carefully tracked their cycles digitally or on paper, enabling planning ahead and pre-preparation. While this was effective for some participants, for example Fran finds comfort knowing that her ovulation date often coincides with low mood, enabling self-reassurance that it will pass, most participants find the inaccuracy of cycle tracking burdensome, for example one participant explained that she would take the predictions very literally and consequently find deviations stressful. Furthermore, an aversion to change was frequently cited, so while reassurance was commonly found in forecasting, for some, the anxiety provoked by alteration to daily routine remains unmitigated.

Fran: One of the major things the app does for me is it tracks ovulation dates and I find my mental health goes really really downhill around my ovulation date so it’s quite useful to predict that. So then if I’m having a really, really bad day and I’m just feeling really low and really anxious and I can look at the app and say oh, right OK, I’m around my ovulation date. That’s probably why, I don’t need to panic that I’m going into another mental health episode.

All participants were taking or had previously taken or considered hormonal contraceptives to gain control of their menstruation. Of those who used birth control, four had done so since their early teens and all found it highly effective. Of the two who did not use birth control, one was keen but unable due to co-morbidities and one was open to in the future. For one, the ability to stop her periods felt life-changing, believing that autistic girls would benefit from open discussion:

Claire: My life changed. It was completely different experience, like I don’t have to worry about that every month. So, I think people who have a very disrupted life because of the periods could benefit from something like that . . . but nobody really discusses that. You know, especially young girls who have like a lot of sensory issues then that’s something that potentially could, you know, be discussed or offered.

No participants disclosed having been offered professional support in managing the impact of menstruation. While those who sought contraceptives had found access non-problematic, no support relating to autism-specific challenges was provided. While some were able to seek support from friends or relatives, others spoke of difficulties relating to fundamentally different experiences shared by neurotypical supports. For some, this led to further masking, and for others initiated feelings of alienation.

Anna: You can say, oh, a lot of people find comfort in exercise and that’s fine, I don’t have a problem with that, but especially when it comes to possibly having a very different sensory experience, or like, executive functioning experience, especially when you give them advice that’s something that will work, and then it doesn’t work, you think well clearly there’s something wrong with me because even advice for everybody doesn’t work.

### Theme 3: Reclaiming orderliness

All participants identified factors that exacerbated their negative experiences of menstruation. The suitability and availability of public bathrooms were identified by most as anxiety-inducing, with some expression that this concern is sufficient to halt occupational participation outside of her home. Furthermore, it was also shared how clothing was a barrier in her workplace until she self-discovered a solution that suited her heightened sensory experience during her periods.

Anna: Usually when I am on my period I don’t really go outside because I don’t want to deal with anything, especially, I live in a city – it’s not easy to access a public bathroom in the city, so I definitely don’t want to deal with that.

Claire: I sort of worked it out, for example those soft sweatpants were the best for me, but obviously when you’re working I’m not gonna go to work in sweatpants. So it was a bit tricky to deal with that then, so I wear leggings under my work clothes so that wouldn’t be in touch with my own skin.

In addition, anxiety surrounding trust in, choice of and side effects associated with use of sanitary products was identified as exacerbating for all participants. While most identified significant sensory distress caused by sanitary products, reluctancy to try alternatives for fear of further discomfort and aversion to change was common. Although some found reassurance in self-conducted research on different products and were able to find a “least bad” option for themselves, another explained how her inclination to take information very literally had induced a phobia of pre-toxic-shock syndrome, which prevented her from trying tampons for most of her young adulthood.

Elle: I grew up in the era when they were when they were talking about periods and really, really pushing the beware of Toxic Shock syndrome, and being autistic, I kind of have a tendency to take that very much to heart and it just instilled this horrific level of fear in me and I was absolutely terrified of it.

Co-morbidities were identified as exacerbators, including but not limited to Ehlers-Danlos syndrome, Dyslexia and Fibromyalgia, and the understanding of both autism and these co-morbidities from both medical professionals and ‘wider society’ was frequently acknowledged. One participant shared how when she sought help for her physical symptoms, she was told that she was over-reacting, and another explained how she struggled to process information provided to her as it was not available in an accessible format.

Fran: They told me I probably over-reacting to pain because I was autistic.

Elle: There’s no point saying, oh well, we’ve provided them with the information, but the information is inaccessible . . . [I’m] mildly dyslexic . . . but I find if text is too small or if it’s in certain fonts, I find it really hard to read.

## Discussion and implications

As evidenced above, menstruation-related challenges understood to be autism-specific were identified by all participants, and often framed against having few (if any) autistic peers around which to make any sense of their everyday experience. Corollary to the sensory problems documented, thus, emerges a core sense of psychological isolation in all accounts given. This can, perhaps, be seen to frame the sometimes-oppositional voice of said accounts.

Topically, specific sensory needs during menstruation were identified as significant daily barriers to participating in self-care occupations, although these were acknowledged by most participants as something they rarely felt they could discuss with others. Rather, the ‘suffer in silence’ motif often persisted. This echoes existing research that emphasises autistic females’ particular propensity for masking their publicly autistic traits (Corscadden and Cassely, 2021). The evidence above, conversely, does not align well with literature that identifies social learning techniques as key for autistic people to develop the skills to manage menstruation (Ballan and Freyer, 2017). The participants herein described an experience very different to that of their neurotypical peers, and that relating to, accessing and applying any advice provided was consequently challenging. This suggests that rather than coaching how to ‘cope’ in neurotypical ways, an autism-informed OT approach focusing on unique symptomology and consequent impacts on occupation would be beneficial. Similarly, the sexual education provided to the autistic individuals participating in this study was routinely identified as a barrier to understanding, accepting, and learning to manage their menstruation. In these terms, teaching about sanitary products from a sensory-informed perspective may have aided in discovering what would have worked for them (Tolan et al., 2016).

The ability to gain control over menstruation-related contexts was cited as critical for all participants, with contingencies beyond their control a persistent source of (sometimes debilitating) anxiety. For example, those able to use hormonal contraceptives to alter their experience did so, but those who could not found this frustrating. Participants often tracked their cycles to predict hormonal fluctuations and associated symptoms, providing control over pre-planning and preparations. Prior research identifying choice and freedom as important contributors to occupational participation (Murthi and Hammell, 2021) further builds a case for autism-informed OT in this domain, prioritising individual autonomy and appreciating expert-by-experience accounts. Furthermore, participants identified menstruation as necessitating increased demand on executive function, often contributing to burn-out and (greater) anxiety. As such, balancing maximum involvement and control over therapy, with consideration of increased demand on executive function becomes vital. Indeed, extant research suggests that preparing for and providing tools to manage such executive function challenges falls both within the duty and ability of OT (Fogel et al., 2020), further indicating that this is an area that could, and prospectively should, be addressed as part of OT practice.

Given the above, however, most of the participants in this study articulated cases whereby healthcare professionals had cursorily acknowledged that there might be links between complex menstrual experiences and autism, with negative implications for quality of life and occupational participation, and yet proceeded to offer no further support. As a (logical) corollary of this, those participants shared feelings that healthcare professionals did not understand them, in turn fostering a sense of marginalisation and a lack of confidence in the healthcare system itself. This further underscores the need for bespoke autism-affirming practice, particularly recognising the diversity within autism, as a moral and ethical requirement for OTs and other healthcare professionals to more fully embrace (Dallman et al., 2022).

### Limitations

In terms of limitations, the findings of this research reflect the experiences of six participants, all of whom were white British nationals, English speaking and with internet access. This inherently restricts the extent to which the findings might speak to a more diverse autistic community. In addition, a number of individuals expressed a desire to participate but found the video-interview format ultimately unpalatable. Therefore, future research utilising a wider range of data-collection methods might similarly provide a more sophisticated understanding of menstruation as experienced by the broad and diverse autistic populace.

## Conclusion

This research has provided an initial insight into the lived experience of menstruation for autistic adults, outlining some challenges in occupational performance faced by those affected. It was found that significant barriers to completing occupations of self-care, productivity and leisure are posed by menstruation. Challenges shared by participants were varied, covering internal struggles, cognitive impacts, occupational restrictions, loss of control and external factors, with exacerbations of the experience noted by all. In these terms, a ‘hidden times three’ history of (at some point) menstruating, being autistic and being undiagnosed/unaware of their autistic identity, became salient for all. This speaks strongly to the literature around masking/camouflaging in autism, and also the general literature around stigma and menstruation. Multiple participants expressed gratitude and found validation in the opportunity to share their experience, demonstrating the emotive nature and value of investigating this topic. A dearth of OT research addressing menstruation experience, and in particular research involving autistic individuals, was identified through a literature review, further indicating a potential gap in understanding. The findings of this study demonstrate that unmet needs exist, illuminating challenges that impact both occupational engagement and participation, with likely consequences of occupational deprivation. As such, future research might further investigate these conclusions from an occupational perspective.

**Key findings**

- Menstruation poses unique and specific challenges in occupational performance for autistic individuals which are largely unaddressed in occupational therapy research and practice.
- Participants described a range of ways in which interaction between their menstruation and autism had negatively impacted their core sense of self, and upon their mental and physical well-being.
- A lack of understanding and support from significant others, including healthcare professionals, was ubiquitously reported, further exacerbating all key challenges.

**What the study has added**

This study enhances understanding of the specific challenges that menstruation can cause for occupational performance among autistic adults, not least as a consequence of sensory overload and amplified experiences of anxiety, cognitively debilitating ‘brain fog’ and socially debilitating concerns about cleanliness. Based on these, key proposals are made regarding how occupational therapy might (and probably should) actively and sensitively address these matters.
